# 3D Printed Formwork for Concrete: State-of-the-Art, Opportunities, Challenges, and Applications

**DOI:** 10.1089/3dp.2021.0024

**Published:** 2022-04-11

**Authors:** Andrei Jipa, Benjamin Dillenburger

**Affiliations:** Digital Building Technologies, Institute of Technology in Architecture, Department of Architecture, ETH Zürich, Zürich, Switzerland.

**Keywords:** 3D printing, formwork, concrete, digital concrete, digital fabrication, advanced manufacturing

## Abstract

Concrete is the most used human-made material in the world, and it is responsible for around 8% of the total greenhouse gas emissions worldwide. Hence, efficient concrete construction methods are one of the main foci of research in architecture, civil engineering, and material science. One recent development that promises to achieve this goal is the use of digital fabrication for building components. Most investigations focus on direct extrusion 3D printing with concrete, which has already been covered in several review articles. Conversely, this article reviews a different approach, which focuses on the indirect digital fabrication of concrete through 3D printed formworks. This approach is under investigation for structural and nonstructural, as well as for on- and off-site applications, with a number of projects having already been built, but a comprehensive review of 3D printed formworks has not yet been compiled to synthesize the findings. This article provides a comprehensive map of the state-of-the-art of five different 3D printing technologies used for the fabrication of formworks so far. The aim is to serve as a fundamental reference for future research, provide a basis for consistent language in this field, and support the development of construction standards. The article further discusses the new geometric possibilities with 3D printed formworks and their potential for making concrete construction more sustainable. In addition, the opportunities and challenges of 3D printed formworks are evaluated in the context of other traditional and digital fabrication tools. A synthetic classification in five functional typologies is proposed and illustrated with 30 representative case studies. Finally, the article concludes with a brief reflection on the role of 3D printing in the broader context of formwork innovation and a possible outlook for this technology.

**Figure:**
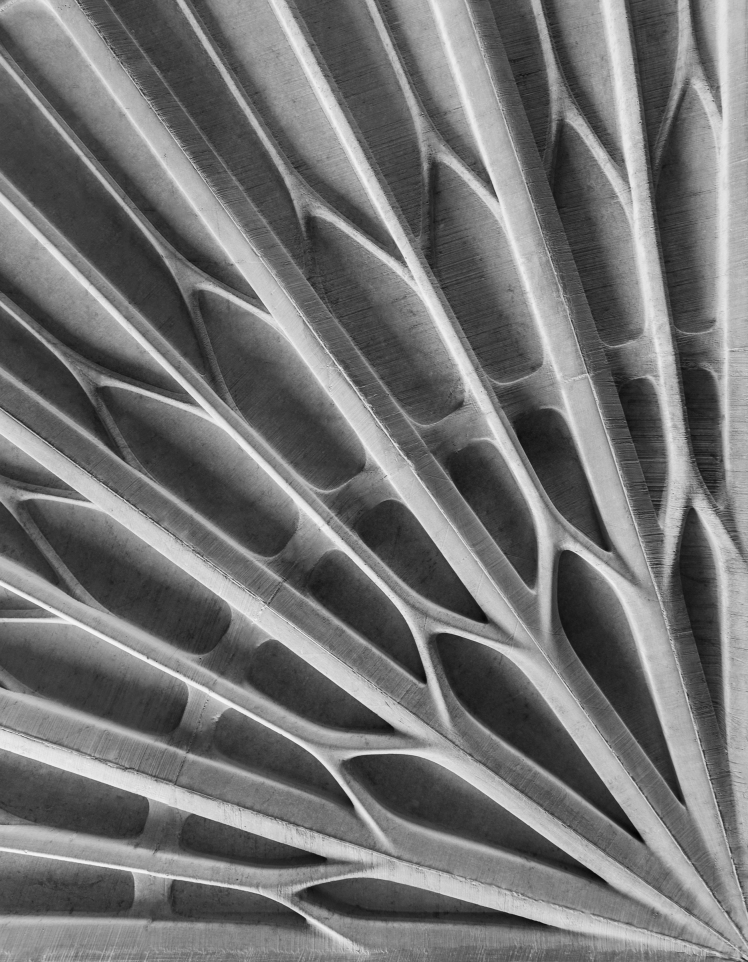


## Introduction

Formworks are the single largest cost component in the construction of a concrete structure.^1^ For this reason, there are currently more than 85,000 patents filed worldwide for inventions concerning formworks for concrete. However, very few consider the use of 3D printing, a fabrication process that has the potential to radically improve conventional formworks in several key respects.

### The role of formwork in concrete construction

Concrete is one of the most sustainable materials available for construction, averaging a specific embodied energy of only 0.75 MJ/kg.^2^ However, concrete is responsible for roughly 8% of the total greenhouse gas emissions worldwide.^3^ This significant contribution is due to the sheer quantity of concrete produced, concrete being the most widely used construction material in the world.^4^

One of the reasons for the wide use of concrete is that the raw materials are readily available locally at low costs in most parts of the world. Due to this, concrete elements are often oversized to keep the construction process quick, simple, and cheap. This paradox of using more material to reduce costs is explained by a fundamental step in the production of concrete: the fabrication of the necessary formwork.

Formwork is the temporary structure used to retain fluid concrete in its designated shape until it hardens. Formworks alone account for more than half the resources necessary for concrete structures, more than cement, aggregates, admixtures, labor, and reinforcement combined. For particular building elements, such as slabs, the formwork can even represent up to 80% of the total costs.^5^ To be more economical, formworks must be simple to fabricate, transport, install, and reuse. Hence, most of the commercially available formwork systems today are based on standardized, mass-produced, reusable, modular flat panels, which put a hard limitation on the shapes that can be achieved in concrete constructions.

Standardized formwork systems often result in oversized, monolithic concrete components. Nevertheless, the overall cost of standard parts is still lower, because the cost of bespoke formworks outweighs the extra cost of raw materials. Furthermore, bespoke optimized geometries incur significantly higher design and engineering costs as opposed to relying on a catalog of standard parts. As a typical example, concrete columns in high-rise buildings are usually sized identically, even if the structurally required section area decreases progressively for upper floors. As a further example, slabs have flat horizontal soffits to be economical. Nevertheless, dropped column heads, ribbed, and waffle slabs are materially more efficient, but can be more expensive to fabricate.

Oversized building elements have a knock-on effect on the substructures of the building. For example, thicker slabs have a larger dead-load and require thicker columns and walls, which, in turn, require deeper foundations. More material means a bigger carbon footprint. Furthermore, the implications do not stop at the construction phase, but impact the end-of-life strategies for the oversized concrete buildings too.

### Digital concrete

To address the challenges of the formwork, two different approaches that involve advanced or digital fabrication are currently being investigated. The first approach looks into the direct digital fabrication of concrete (DDFC), which completely eliminates the need for formwork. The second approach looks into the digital fabrication of formworks (DFF), which radically expands the geometric possibilities with concrete.

DDFC has been covered in recent years by extensive reviews,^6,7^ as well as by articles focusing on specific methods.^8^ Wangler *et al.* identify three types of DDFC: layered extrusion, slip forming, and binder jetting.^6^ These processes share several challenges that currently prevent them from being used in structural applications.^9^ The main limitation of DDFC concerns the reinforcement. While short fiber reinforcement is possible to a certain extent, the inability to integrate continuous rigid reinforcement bars is a significant penalty for the bending strength of DDFC. This limitation, along with the material discontinuity inherent in layer-based fabrication processes, prevents DDFC from being certified for compliance with the statutory requirements of building standards.

Furthermore, even if DDFC does not require formworks, it still has significant geometric constraints, limiting build heights and volumes, cantilevers for the extrusion-based processes, inner voids for binder jetting, and resolution for slip forming. DFF aims to address these limitations.

### Digital formworks

At the end of the 19th century, the early attempts to break away from the constraints of the flat panel formwork used patterning and stitching of flexible materials to create new concrete shapes. Flexible materials such as geotextiles can be used for ultra-light developable formworks.^10^ Recent research has shown that flexible, lightweight formworks can have nondevelopable shapes as well, through numerically controlled knitting.^11,12^

Pneumatic formworks are another one of the earliest methods for creating nonstandard concrete shapes, especially long-span shells. It relies on flexible membranes that can be inflated either before or after the concrete is applied. The method was introduced in the 1920s and it became more prominent in the 1970s and 1980s with the work of Heinz Isler and Werner Sobek.^13,14^ Inflatable formworks have also been used in conjunction with robotic fabrication for a thin carbon fiber bespoke shell with a span of 8.5 m.^15,16^ This suggests that the limited geometric possibilities of pneumatic formworks for concrete can also be expanded through the use of digital fabrication.

Further geometric freedom can be achieved with subtractive fabrication processes. Computer numerically controlled (CNC) hot wire cutting of extruded polystyrene blocks is one of the most used commercial methods to fabricate formworks with nonstandard shapes.^17^ Variations of this method, such as robotic hot-wire cutting,^18^ hot blade cutting,^19^ and spatial wire cutting,^20^ have been used to extend the geometric capabilities of CNC wire cutting beyond ruled surfaces. CNC cutting of flat sheet materials such as plywood and plastics has also been actively used to create moderately curved concrete shells.^21^

Formative fabrication is another category of processes that is intensely researched and occasionally used in building projects. Processes such as CNC thermoforming and molding of glass-fiber-reinforced polymers (GFRP) produce reusable formworks, but with significant geometric constraints. Land forming is another process in the formative fabrication family, which has only been used experimentally for the fabrication of formworks so far. It relies on casting, spraying, or extruding concrete on a mechanically or robotically shaped reusable granular substrate, such as earth, sand, or gravel.^22–24^ CNC folding of thin sheet materials has been successfully used to produce minimal plastic formworks.^25^

Another formative process, Mesh Mold, produces a functional stay-in-place formwork by robotically bending, cutting, and welding steel reinforcement rods on-site.^26^ A final example of formative fabrication are actuated molds that deform a thin sheet of lining material, either before or after the concrete placement.^27,28^

In contrast to DDFC, which eliminates formworks altogether, all the DFF processes rely on formworks, the most expensive aspect of concrete construction. Nevertheless, formworks have in this case their own advantages. DFF allows the precise control of the concrete surface quality, does not require special concrete mixes, and allows the use of regular rigid reinforcement. DFF relies on conventional concrete casting techniques that are familiar to the building industry and can be easily certified for the use in constructions. However, all the processes described so far still have geometric limitations. High-resolution details and surface features are generally not easily achievable. Most methods have process-specific limitations: ruled surfaces for CNC hot-wire cutting, developable surfaces for CNC folding, anticlastic surfaces for fabrics, undercut-free shapes for milling, and limited principal curvatures for formative processes.

Therefore, despite all the advances in DFF, the geometric potential of concrete is still latent due to the various limiting factors, such as tool accessibility (e.g., undercuts for CNC milling), material constraints (e.g., behavior of fabrics), and economic constraints (e.g., wasteful and time-consuming high-resolution milling). These geometric limitations can be overcome by using 3D printing for the fabrication of formwork. While not fully unconstrained geometrically, 3D printing can produce significantly more complex geometries with higher precision and resolution.^8^

## State-of-the-Art

**FIG. 1.**
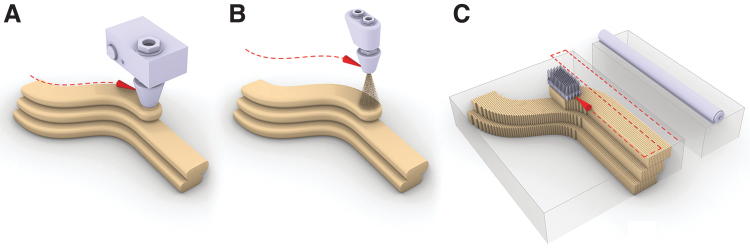
The three different types of 3D printing processes that are being used for the fabrication of formworks for concrete: **(A)** material extrusion with concrete, clay, or polymers; **(B)** material jetting with wax or foam; **(C)** binder jetting (DBT, ETH Zürich, 2021).

3D printing is an umbrella term that covers a range of different processes as defined in ISO 52900.^29^ Out of these, binder jetting, material extrusion, and material jetting have been used so far for the fabrication of formworks (Fig. 1). Table 1 summarizes these 3D printing processes and their corresponding materials and consolidation mechanisms used for 3D printed formworks (3DPF).

**Table 1.** A Summary of the Five Different Types of 3D Printing Technologies Used for Formwork Fabrication and the Process of Joining or Solidifying Particles they are Based on

Process	Material extrusion			Material Jetting		Binder-jetting
Material	Concrete	Clay	Polymers	Wax	Foam	Sand + Binder
*Consolidation mechanism*	Chemical hydration	Shear-thinning rheology	Phase transition (liquid–solid)		Chemical polymerization	Adhesive bonding

### Material extrusion

In material extrusion 3D printing, a liquid or paste is dispensed through a nozzle or orifice according to a 3D model (Fig. 1A). The deposition happens usually on a substrate, in consecutive horizontal layers that are generated as slices through a digital model, but spatial extrusion following nonlayered toolpaths is also possible with clays and polymers.^30^ The consolidation process of the deposition differs depending on the material used: chemical hydration for cementitious materials, shear thinning rheology and drying for clays, and freezing or liquid to solid phase transition due to cooling for thermoplastic polymers.

#### Concrete extrusion

##### Process description

In concrete extrusion, a cementitious paste is extruded. The structural buildup of the deposition comes from the chemical hydration of the Portland cement in the mix. A critical aspect for achieving large scale with this process is the precise control of the material rheology. Concrete needs to be pumpable until it reaches the nozzle and then accelerated to quickly achieve enough strength to support subsequent layers. State-of-the-art research is currently investigating the replacement of Portland cement with more sustainable geopolymers and the integration of fiber reinforcement in the mixes.^31,32^

##### Implementations

**FIG. 2.**
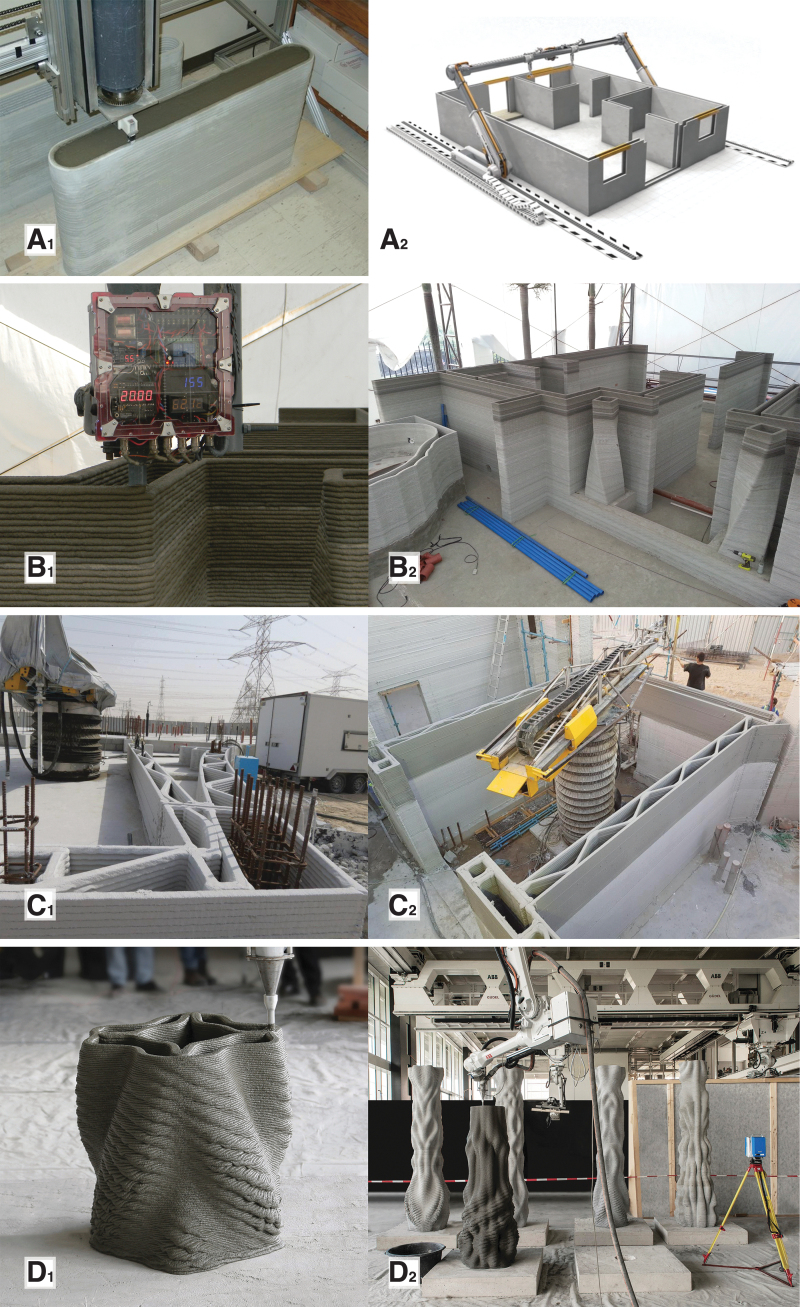
An overview of some of the direct digital fabrication of concrete processes used for fabricating formworks for concrete: **(A_1_)** the contour crafting gantry and tool-head producing the formwork for a prototypical wall segment that is subsequently filled with concrete (© Contour Crafting Corporation, used with permission); **(A_2_)** the proposed *in situ* fabrication setup of contour crafting, showcasing a rail-mounted concrete printing gantry (© Contour Crafting Corporation, used with permission). **(B_1_)** The gantry-mounted tool head of the Total Kustom printing system (© Total Kustom, used with permission); **(B_2_)** the system was used for the *in situ* fabrication of a hotel suite in the Philippines, including two twisted columns with nonstandard geometry. Concrete extrusion printing was used to create hollow thin shells that acted as stay-in-place formworks for the vertical load-bearing elements (© Total Kustom, used with permission). **(C_1_)** Detail of a 640 m^2^ administrative building in Dubai, fabricated using *in situ* 3D printed concrete formworks by Apis Cor in 2019. Some of the open cells of the cellular wall structure contain steel reinforcement cages and are subsequently filled with concrete (© Apis Cor, shared media package); **(C_2_)** the *in situ* fabrication setup of Apis Cor, featuring a deployable polar robotic system (© Apis Cor, shared media package). **(D_1_)** Detail of the prefabrication of one of the columns of Concrete Choreography, a project developed at DBT, ETH Zürich for a performing arts festival in Graubünden, Switzerland, in 2019. **(D_2_)** Unlike examples A, B, and C, Concrete Choreography proposes a prefabrication process for 3D printed concrete formworks, using the setup in the Robotic Fabrication Laboratory of ETH Zürich, using a robotic arm mounted on a suspended gantry (© Axel Crettenand/DBT, ETH Zürich).

Fig. 2 presents a selective overview of significant projects that use concrete extrusion for 3DPF. Concrete extrusion is the only process reviewed in this article that was developed from the beginning as a formwork fabrication process. The pioneering contour crafting process, initially developed in 1995, is based on the extrusion of discrete layers of cementitious material to form an enclosed shell that subsequently acts as lost formwork for casting concrete.^33^ The system is designed for on-site construction of simple vertical or slightly inclined concrete walls.^34^ The process included forward-looking features such as the embedding of reinforcement between layers, and an integrated troweling tool for leveling out interlayer gaps to achieve a smooth surface. Another idea put forward in contour crafting was the sequential casting of concrete in parallel with the 3D printing of the concrete formwork. This idea was later developed by projects such as Eggshell and Yhnova to mitigate hydrostatic pressure buildup. Finally, contour crafting theorized the integration of robotically placed rigid reinforcement and building services, as well as the provision of 3D printed tubes for post-tensioning tendons. About 20 years after its initial development, instances of the contour crafting technology started to be used in commercial constructions by companies such as Winsun, Huashang Tengda, and Total Kustom.^35^ These companies pursued similar approaches, in which a concrete shell is 3D printed, steel reinforcement cages are added, and finally concrete is cast.

Contour crafting was further adapted and implemented in 2017 by XtreeE for a 4-m-high load-bearing column designed by EZCT in 2015. The column was prefabricated for a school in Aix-en-Provence, France, designed by Marc Dalibard.^36,37^ The column was topologically optimized and prefabricated in four distinct elements that were assembled on-site. Unlike contour crafting, which trowels the wet concrete as it is printed to make it smooth, the Aix-en-Provence column was mechanically processed and polished to erase the traces of layers only after the concrete cured. The same method was further used in a different application, for one-of-a-kind concrete façade panels and a 2.5-m-long wall for the YRYS Concept House.^38^

Another company, Apis Cor, uses local casting inside 3D printed cellular walls around manually placed steel reinforcement bars.^39^ The company uses deployable polar concrete 3D printing robotic arms, and has demonstrated the technology at 1:1 scale for small prototypical housing units, as well as for a 640 m^2^ administrative building in Dubai, in 2019. Apis Cor has made significant efforts getting their technology to meet local building standards, by benchmarking it against conventional methods such as concrete masonry unit walls.

In 2019, concrete 3DPF were used for Concrete Choreography, a series of 12 three-meter tall columns commissioned by a performing arts festival in Riom, Graubünden, Switzerland. The fabrication process involved the following steps: first, a thin double concrete shell was fabricated using robotic concrete 3D printing; a steel reinforcement cage and post-tensioning tendon were inserted in the internal void of the columns; and finally, concrete was cast in the void around the reinforcement cage. Hence, the 3D printed concrete shell acted as stay-in-place reinforcement. The columns showcase a higher geometric complexity than previous projects, with cantilevers, undercuts, voids, and inner chambers.^40^

#### Clay extrusion

##### Process description

In clay extrusion, a ceramic slurry or paste is pushed through a numerically controlled deposition tool. It is essentially similar to concrete extrusion, but the structural buildup is achieved due to the non-Newtonian shear-thinning rheology of the extruded paste: once the shear-inducing extrusion process ceases, the viscosity of the material increases. The printed parts are typically very fragile and soft and usually rely on a postprocessing stage like drying or sintering to achieve the necessary mechanical properties.

##### Implementations

The process was first developed in the 2000s, and unlike concrete extrusion, it was not intended initially for formworks.^41,42^ One advantage for which clay extrusion started to be used in formwork fabrication is the lack of water resistance of clay, which makes it easy to wash away for demolding.

In 2015, XtreeE used 3D printed clay formworks for the first time to produce a prototypical excerpt of a custom space truss designed to reduce the amount of material using a genetic algorithm.^43^ The resulting concrete surface inherited the textured mold resulting from the layer-based process, and subsequently, the truss was mechanically smoothened. In the following year, this technique was the focus of a master's design studio in London.^44^ The students printed disposable clay formworks for concrete objects of up to 2.5 m in height. The material rheology was controlled by adjusting the amount of water in the clay paste, and limited cantilevers were also achieved.

#### Polymer extrusion

##### Process description

Polymers are widely available materials for extrusion 3D printing. The process first heats and melts the material, which is extruded in liquid state, and subsequently solidifies immediately after the deposition due to cooling. A wide variety of plastics can be extruded, including biodegradable, water-soluble, fiber-reinforced, flexible, conductive, low-shrinkage, or bio-based polymers.

**FIG. 3.**
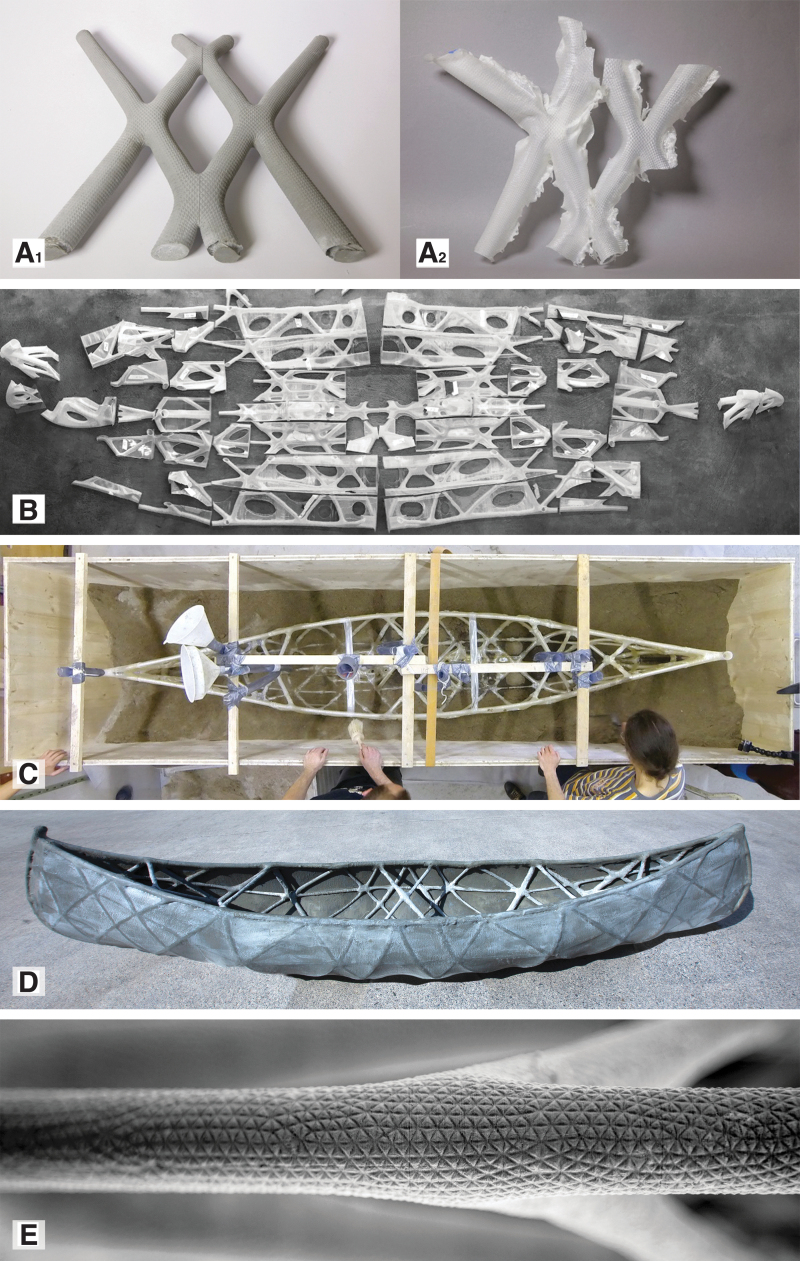
SkelETHon, the 4-m-long concrete canoe. **(A_1–2_)** For the project, a series of small prototypes was initially used to develop an adequate concrete mix that can fill narrow and long tubular structures with widths between 12 and 20 mm. **(B)** The canoe was then fabricated based on 84 discrete 3D printed biopolymer formwork parts with a thickness of 0.8 mm. **(C)** To prevent the hydrostatic pressure from breaking the formwork during casting, the formwork was submerged in sand. **(D, E)** The thin formwork was easily removed using a heat gun and the resulting concrete surface featured a precise texture with submillimeter details (PCBM, DBT, ETH Zürich, 2018).

Furthermore, despite some fabrication limitations, such as unsupported cantilevers, polymer extrusion is unique for its capability to produce in large-scale freestanding shells with thin geometric features and walls less than 1 mm in thickness (Fig. 3). Polymer extrusion is a versatile, accessible, and cost-efficient process, available not only in small desktop machines but also in large robotic gantry setups of up to several hundred cubic meters. Polymer extrusion is known commercially as fused deposition modeling (FDM) or fused filament forming.

##### Implementations of spatial extrusion

**FIG. 4.**
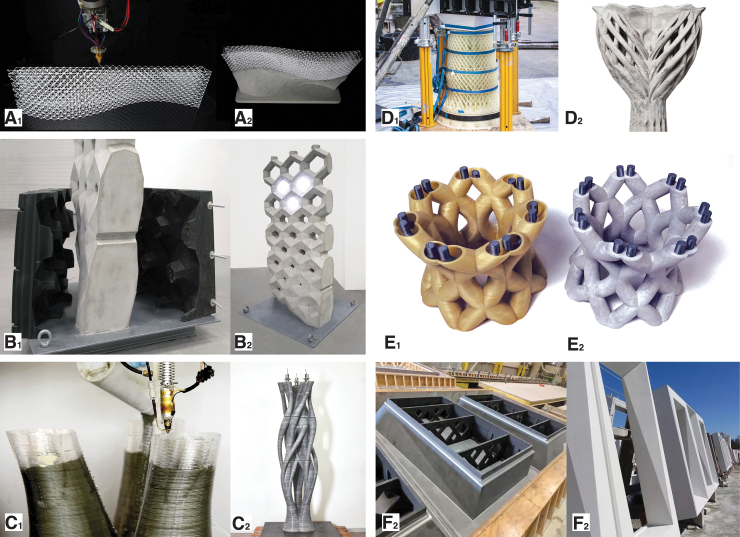
An overview of some of the projects that use polymer extrusion for the fabrication of formworks for concrete: **(A_1–2_)** The spatially extruded polymer lattice proposed by Mesh Mold, which acts as a formwork and secondary reinforcement for a free-form wall prototype (© Gramazio Kohler Research, ETH Zürich, used with permission). **(B_1–2_)** The heavy-duty 3D printed polymer formwork for the NOWlab functional wall with integrated lighting features (© NOWlab, 2018; used with permission of Immensa Labs). **(C_1–2_)** The Eggshell process, which focuses on the 3D printing of a minimal plastic shell and simultaneous concrete casting (© Gramazio Kohler Research, ETH Zürich, used with permission). **(D_1–2_)** The dissolvable formwork for a structural *column* with a free-form capital developed at DBT, ETH Zürich (© DBT, ETH Zürich). **(E_1–2_)** The dissolvable formwork for a small concrete prototype developed at Iowa State University (© Shelby Elizabeth Doyle/Iowa State University, used with permission). **(F_1–2_)** The reusable ABS formwork, printed using the Big Area Additive Manufacturing facility, used for the concrete façade panels of the high-rise apartment building in the Domino Sugar Refinery complex in New York (© Gate Precast Company, used with permission).

Fig. 4 presents a selective overview of some projects that use polymer extrusion for 3DPF. Polymer extrusion was first developed in the late 80s and patented in 1992.^45^ The first use of polymer extrusion for 3DPF was for Mesh Mold, a project developed in 2013.^46^ The method spatially extruded a thermoplastic lattice structure that acted both as formwork and secondary reinforcement. The idea of using polymer extrusion for formwork lattices was subsequently developed further and patented as Cellular Fabrication by a different team in 2017.^47^ Spatial extrusion has also been used in combination with layer-based polymer extrusion for the fabrication of a concrete wall prototype. The system proposed a thin 3D printed layer-based formwork lining supported by a structural spatial mesh 3D printed in parallel.^47^

Nonlayer-based polymer extrusion can also be achieved in combination with other fabrication methods, such as with patterned fabric formworks. It is possible to 3D print directly on textile materials, such as spacer fabrics, to control their behavior and integrate a patterned ornament on the concrete surface.^48^

##### Implementations of layer-based extrusion

A patent for using a more conventional layer-based polymer extrusion 3D printing for flexible formworks was filed in 2014. The proposed method, named Additive formwork, was demonstrated in a series of small-scale prototypes.^49,50^ Layer-based polymer 3DPF were developed further in 2016, in a research for Submillimeter formworks.^51^ Submillimeter formworks were used for a 4-m-long functional concrete canoe (Fig. 3).^52^ An innovation of polymer 3DPF was proposed independently by two different teams from Iowa State University and ETH Zürich in 2019. The innovation focuses on water-soluble polyvinyl alcohol (PVA) for fabricating easily removable and fully recyclable formworks for columns.^53–56^

**FIG. 5.**
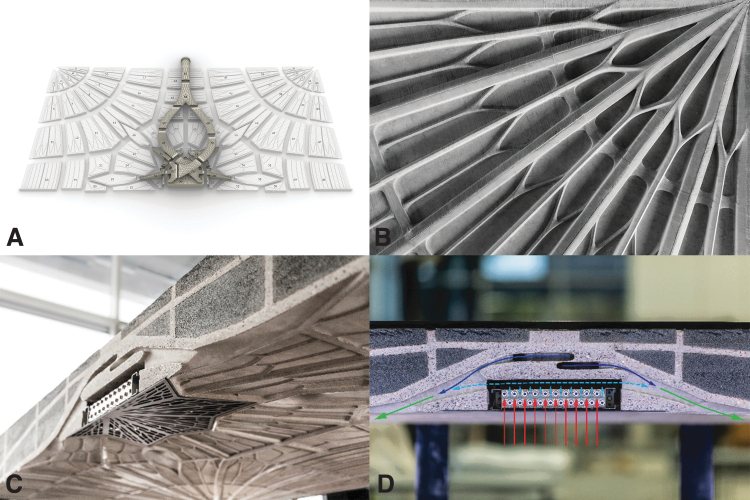
**(A)** The surface of the Funicular Slab was defined by 42 discrete submillimeter 3DPF reinforced by a diagrid of ribs and welded for casting. **(B)** The soffit exhibits a complex compression-only structural system showcasing a finely ribbed surface reminding of a gothic tectonic. **(C, D)** The 3D printed polymer formwork also defines a complex heating, ventilation, and air conditioning system of ducts integrated directly into the concrete slab (© BRG, A/S, DBT, ETH Zürich, 2016).

Another innovation was proposed in Eggshell formworks, which are based on a simultaneous casting and 3D printing process to tackle the challenges of the fragile Submillimeter formworks developed earlier.^57^ Submillimeter formworks were also used to fabricate the Funicular Slab prototype for the World Economic Forum 2020 in Davos (Fig. 5).^58^ The slab integrates within its structural depth a highly optimized complex network of heating, ventilation, and air conditioning (HVAC) air ducts formed with 3DPF. MAS Stairs is a project based on two master students' theses, who used submillimeter polymer 3DPF to produce large concrete structures in discrete prefabricated segments. MAS Stairs focused on structurally optimized post-tensioned stairs assembled from precast individual steps.^59^

Instead of minimal disposable formworks, another approach to achieve material efficiency proposes a robust, heavy-duty formwork system that can be reused multiple times. The method was used by NOWlab and Immensa Labs for a conceptual prototype of a functional wall with integrated lighting.^60^ Later, NOWlab used the same approach for a refurbishment project, in which arched lintels were cast in concrete based on reusable 3DPF.^61^ Furthermore, in 2018, a patent was filed by BigRep for using this method for the fabrication of a flat-panel 3DPF system structurally optimized according to the hydrostatic load.^62^ This development goes back toward the standardized flat-panel formwork systems that were initially challenged by 3DPF.

A similar approach, aiming to 3D print robust and reusable formworks, was also implemented with the Big Area Additive Manufacturing (BAAM) facility for a high-rise apartment building in the Domino Sugar Refinery complex in New York.^63^ BAAM was able to reuse formworks up to 190 times with minimal maintenance work.

Several other projects addressed new, experimental approaches to 3DPF. The Vulcan Pavilion used small prefabricated polymer 3DPF modules that were partly filled with concrete to increase the stability and integrate an opacity gradient in a large-scale vaulted structure.^64^ A different approach was taken by a Aectual, a Dutch company based in Amsterdam, relying on robotic 3D printing of complex 2D patterns for terrazzo floors. Aectual also experimented with polymer extrusion for a stair.^65^ Another recent project from CREATE, University of Southern Denmark, investigated the fabrication of informed textures with polymer 3DPF and measured the deviations of the resulting concrete parts and formworks compared to the designed digital model.^66^

### Material jetting

Material Jetting is an additive manufacturing process in which drops of feedstock material are sprayed by one or more nozzles on a substrate from a distance (Fig. 1B). The spraying generally follows consecutive horizontal layers that are generated as slices through a digital model. A first instance of the process was originally patented in 1999, under the name of PolyJet. PolyJet used inkjet printheads with nozzles less than 1 mm in diameter to spray photopolymers that harden when exposed to ultraviolet light. These photopolymers can achieve excellent resolutions, but are not being used for fabricating formworks. Nevertheless, this process has been scaled up to operate with different materials such as wax and foam and nozzles with significantly larger diameters.

#### Wax jetting

##### Process description

The process involves a hot wax in liquid state that is jetted onto a support surface from a relative distance. The distance controls a cone of dispersion and implicitly the width of the deposited material bead. The structural buildup of the deposition comes from the change in phase of the wax material from liquid to solid. The waxes used in this process have relatively low melting points 40–70°C, require little energy to be melted, and transition quickly back to the solid state at room temperature.

##### Implementations

3D printed wax was proposed as a solution for achieving zero-waste formwork due to its potential to be recycled with little energy and reused multiple times.^67^ The method, called FreeFab, uses wax jetting for achieving the rough outline of the formwork, which is subsequently milled to a precise tolerance using a rotating numerically controlled spindle.^68^ The wax is soft, requiring little energy to be milled, while the subtracted material can be easily recyclable. The concrete is sprayed and the smooth milled surfaces of the formworks facilitate an easy demolding process. After the concrete cures, the wax can be crushed, melted at low temperatures, filtered, and reused. FreeFab was used for a series of infrastructure projects in London: Tottenham Court Road, Bond Street, and Liverpool Street. Around 1400 unique wax formwork panels were made using FreeFab for the bespoke geometry of the prefabricated concrete lining for the underground tunnels.^69^

#### Foam jetting

##### Process description

Foams constitute another class of materials used in material jetting, as well as in extrusion 3D printing. They are dispensed through a numerically controlled nozzle in a liquid state and a blowing agent triggers the foaming process through the formation of gas bubbles. The stabilization of the deposited foam is a result of chemical cross-linking that increases the viscosity and eventually leads to solidification. By controlling the blowing agent, foams with different properties can be obtained, from soft to tough, from elastic to rigid, and from lightweight foams with large open pores to foams with fine dense closed pores. These properties can be useful in creating architectural qualities such as transparency or insulation properties.

##### Implementations

Spray Foam is a foam jetting 3D printing method patented in 2013. The patent describes a nozzle that sprays foam layer by layer, followed by a subtractive tool that corrects the layer irregularities to fabricate an object according to a CAD model.^70,71^ According to the patent, the main application for this compound fabrication method is to produce formworks for concrete walls in residential buildings.

This research on foam 3D printing was continued in the direction of *in situ* fabrication of lightweight large-span structures, but the idea of using it as formwork for concrete was abandoned. In 2016, a similar technique to Spray Foam was demonstrated for the fabrication of the walls for a social house in Nantes, France. The new system, named Batiprint3d™, proposed the use of the same robotic arm to 3D print the foam and to extrude the concrete infill.^72^

### Binder jetting

#### Process description

In binder jetting (BJ), a liquid binder is selectively applied on successive thin layers of powder (Fig. 1C). Its significant advantages are its high resolution, geometric freedom, and large volume. Binder jetting can theoretically be used with any powder material that can be bonded, such as cement, geopolymers, plastics, ceramics, metals, sand, sugar, and plaster.^73^ Moreover, this process has the advantage that geometric complexity is decoupled from printing time and production cost. On the downside, the process often requires time-consuming postprocessing steps for cleaning loose powder and stabilizing the printed parts, such as microwave curing or surface infiltration treatments.

#### Implementations

**FIG. 6.**
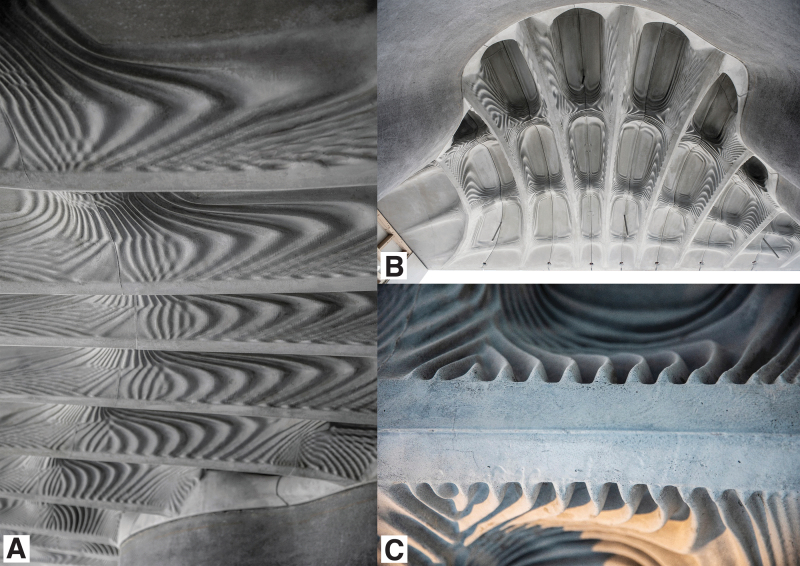
**(A, B)** Details of the 11 similar, but customized ribs of the Smart Slab, with an optimized macrogeometry adapted to the support conditions. **(C)** Detail of the millimeter precise features, fabricated using binder-jet disposable 3DPF (© DBT, ETH Zürich, 2018).

**FIG. 7.**
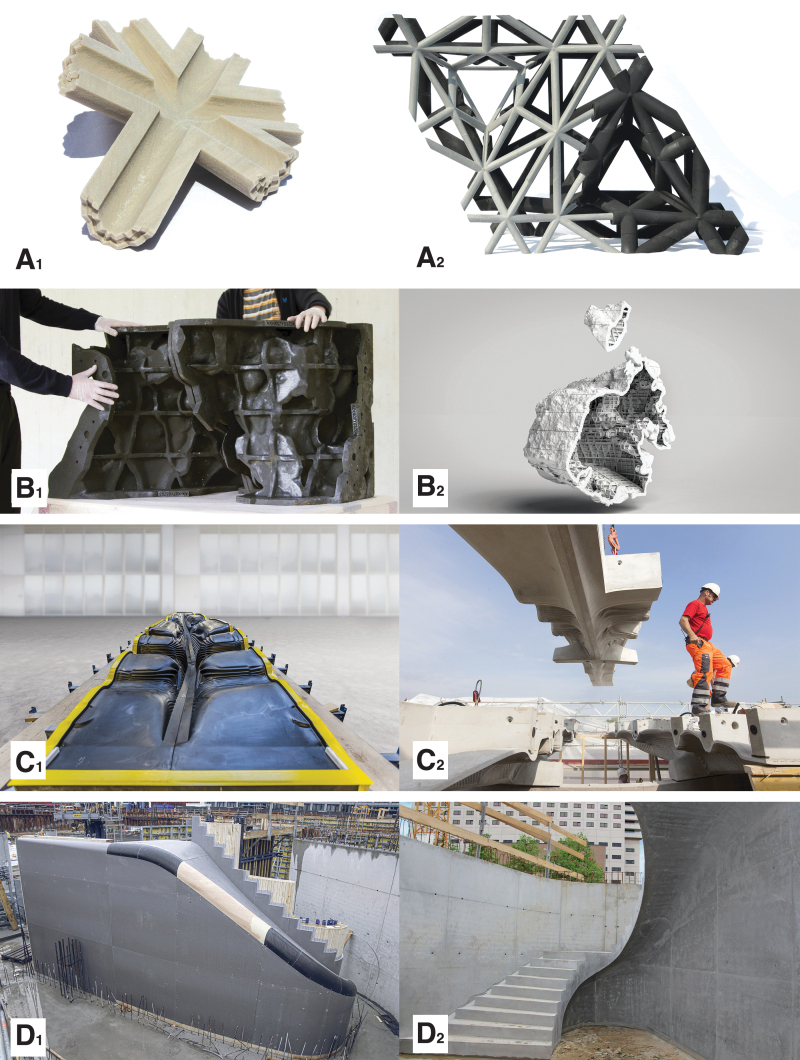
An overview of some of the projects that use binder jetting for the fabrication of formworks for concrete: **(A_1_)** a piece of binder-jet formwork part, 3D printed by Voxeljet for one of the complex nodes of the Studies in Recursive Lattices project by EZCT Architecture & Design Research, 2012–2013; **(A_2_)** The formwork was filled with Ductal^®^ ultra-high-performance concrete by Fehr GmbH and was partly demolded to be presented in Frac Centre, Orléans at the 9th ArchiLab exhibition entitled Naturalizing Architecture. The prototype is currently part of the Centre Pompidou permanent collection (© EZCT Architecture & Design Research, 2012–2021). **(B_1_)** Two formwork parts, 3D printed using binder-jet technology by Christenguss AG, for the Incidental Space, the Swiss pavilion at the Venice Biennale 2016. The formwork parts exhibit a geometric stiffening grid of ribs on the back side (© Christian Kerez/DBT, ETH Zürich, 2017). **(B_2_)** A computer-generated sectional view of the formwork for the pavilion designed by Christian Kerez and digitally fabricated by DBT, ETH Zürich (© Christian Kerez/DBT, ETH Zürich, 2017). **(C_1_)** Fourteen 3D printed parts fabricated by Voxeljet, assembled to form the formwork for one of the prefabricated segments of the Smart Slab, a research project led by DBT, ETH Zürich (© DBT, ETH Zürich). **(C_2_)** The on-site assembly of the precast segment, concreted by Bürgin Creations, using spraying and casting of lightweight polymer-fiber-reinforced concrete (© DBT, ETH Zürich). **(D_1_)** View of the formwork for the stair of the Neubau Sächsische Aufbaubank in Leipzig, designed by Acme. The formwork not only uses mainly standard Doka systems but also a series of small 3D printed segments, seen in *black* in the image. These segments, produced by Voxeljet using binder jetting, interface seamlessly with the standard formwork system (© Doka, shared press package). **(D_2_)** View of the finished staircase (© Doka, shared press package).

**FIG. 8.**
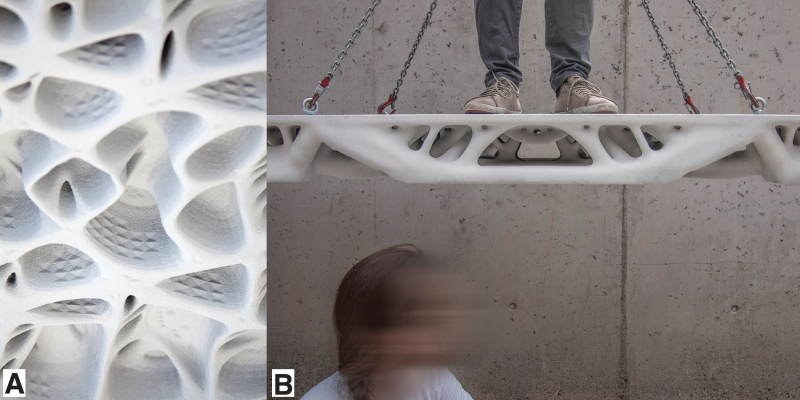
**(A)** The soffit of one of the two Smart Take from the Strong slab prototypes exhibiting an articulated surface that reflects the optimized topology and a finer, secondary texture proposed for improving acoustic properties in a specific range of frequencies. **(B)** The optimized thickness of the second Smart take from the Strong slab prototype, achieving a 70% material reduction compared to a conventional concrete slab. The two prototypes were fabricated using a 9-mm-thick stay-in-place binder jet 3DPF (DBT, ETH Zürich, 2017).

Fig. 6, 7 and 8 present a selective overview of projects that use binder jetting for 3DPF. The first research project using this method was Studies in Recursive Lattices.^74^ The formwork consisted of 252 binder-jetted parts, coated and assembled to create the 1.9 × 1.6 × 0.9 m structural lattice that optimizes the use of high-performance concrete.

A step up in scale was the Incidental Space by Christian Kerez, the Swiss pavilion at the Venice Biennale in 2016.^75^ The 9-m-long by 6-m-high space was defined by a thin convoluted shell of fiber-reinforced shotcrete with a thickness varying between 10 and 40 mm. Around a third of the 2000 formwork parts were 3D printed with a large-scale binder-jet printer. In the same year, The Smart Take From the Strong, a pair of concrete slab prototypes measuring 1.8 × 1 m showcased how stay-in-place 3DPF can enable the fabrication of topologically optimized building elements.^76^ Two years later, the prototypes were followed up by the Smart Slab, which used a disposable formwork system to prefabricate a 15-tonne cantilevering structural slab (Fig. 7).^77^ The 206 unique formwork parts enabled the fabrication of a computationally optimized and efficiently post-tensioned slab that weighs 65% less than a conventional concrete slab.

A formwork system for *in situ* casting was proposed later the same year for fabricating a winder stair for the headquarters of the Sächsische Aufbaubank in Leipzig, Germany.^78^ 3D printing was used minimally, only to define the nondevelopable lining of the stair intrados, where standard plywood formwork was inadequate for defining the geometry. Finally, in 2020, Fast Complexity, a system combining reusable binder-jet 3DPF and DDFC, was proposed for prefabricated discrete post-tensioned slab elements.^79^

## Classification of 3D Printed Formwork Strategies

According to the fabrication method, concrete can be classified in cast, sprayed, extruded, slip formed, and, more recently, 3D printed, either through extrusion or binder jetting. Binder jetting, also referred to as particle-bed binding, is the only concrete 3D printing process in which formworks are not used.^9^ 3DPF have not yet been used for extrusion and slip forming, processes in which a mold supports only a discrete section of the concrete structure and gradually slides to form the subsequent sections. The remaining three processes, casting, spraying, and extrusion 3D printing, have all been tested for 3DPF.

Formworks have a significant influence on concrete construction, through specific site logistics and building sequences. Concrete constructions are either prefabricated in discrete elements off-site and assembled on-site or cast monolithically *in situ*. A combination of partial prefabrication and *in situ* casting is also possible. Formwork is always prefabricated and usually preassembled in modules that are put together on site. Sometimes, the logistical advantages of *in situ* concrete are negated by the need to prefabricate, transport, and assemble the formwork. To address this, 3D printed foam and concrete formworks introduce the novel concept of *in situ* formwork, enabling better coordination with the *in situ* concrete casting. The concept of simultaneous formwork fabrication and concrete casting pushes this idea a step further and can prove to be a powerful tool in on-site construction.^80^

Depending on what happens to the formwork once the concrete cures, two main categories can be defined: removable and stay-in-place. Five further subtypes are discussed in Table 2 in relation to a series of example projects and the concreting method.

**Table 2.** An Overview of the Projects Reviewed in this Article, Classified by Typology, 3D Printed Formworks Technology, Reinforcement Type, and On- or Off-site Construction

Type	Project	3DPF	Element	Formwork	Concrete		Reinforcement
**Removable**							
Reusable	Aufbaubank Stair	BJ	Stair	Prefab	Cast	*In situ*	Rebar
	Fast Complexity	BJ	Slab	Prefab	Printed	Prefab	Hybrid
	Domino Sugar Refinery	FDM	Facade	Prefab	Cast	Prefab	Fibers
	Additive Formwork	FDM	Wall	n/a	Cast	n/a	n/a
	NowLab Wall	FDM	Wall	Prefab	Cast	n/a	n/a
Disposable	Recursive Lattices	BJ	Space frame	n/a	Cast	n/a	Fibers
	Smart Slab	BJ	Slab	Prefab	Sprayed	Prefab	Hybrid
	Incidental Space	BJ	Freeform	Prefab	Sprayed	Prefab	Fibers
	Submillimeter formwork	FDM	Column	Prefab	Cast	Prefab	Fibers
	Water-soluble formwork (ETH)	FDM	Column	Prefab	Cast	Prefab	Fibers, rebar
	Dissolvable formwork (Iowa)	FDM	Column	Prefab	Cast	n/a	3D printed
	Eggshell formwork	FDM	Column	Prefab	Cast	Prefab	Prestressing
	MAS stairs	FDM	Stair	Prefab	Cast	Prefab	Prestressing
	FreeFab Wax formwork	FDM	Façade	Prefab	Sprayed	Prefab	Fibers
	Concrete Canoe	FDM	Space frame	Prefab	Cast	Prefab	Fibers
	XtreeE Truss	Clay	Space frame	Prefab	Cast	Prefab	Fibers
	UCL Clay formwork	Clay	Prototypes	Prefab	Cast	Prefab	n/a
**Stay-in-place**							
Structural	Mesh Mold	FDM	Walls	*In situ*	Sprayed	*In situ*	None
Functional	HVAC						
	Funicular Floor Slab	FDM	Slab	Prefab	Cast	Prefab	None
	Insulation						
	Spray Foam Wall	Foam	Wall	*In situ*	Cast	*In situ*	n/a
	Yhnova Foam formwork	Foam	Wall	*In situ*	Cast	*In situ*	n/a
	Reinforcement cover						
	Aix-en-Provence Column	Concrete	Column	Prefab	Cast	Prefab	Fibers
	Concrete Choreography	Concrete	Column	Prefab	Cast	Prefab	Rebar
	Contour Crafting	Concrete	Walls	*In situ*	Cast	*In situ*	Rebar
	Huashang Tengda	Concrete	Walls	Prefab	Cast	Prefab	Rebar
	Total Kustom	Concrete	Walls	*In situ*	Cast	*In situ*	Rebar
	WinSun	Concrete	Walls	Prefab	Cast	Prefab	Rebar
	YRYS Concept House	Concrete	Wall	Prefab	Cast	Prefab	n/a
	Apis Cor	Concrete	Walls, slabs	*In situ*	Cast	*In situ*	Rebar
Permanent	Smart Take from the Strong	Binder Jet	Slabs	Prefab	Cast	Prefab	Fibers

BJ, binder jetting; FDM, fused deposition modeling; HVAC, heating, ventilation, and air conditioning; n/a, not applicable.

### Reusable formwork

Reusable formworks are the most common type of formwork used for standard concrete geometries, relying on heavy duty modular plastic, metal, or timber panels, sometimes with additional lining layers. These formworks can be regularly reused several hundred times with minimal maintenance work.

3D printed reusable formworks have been proposed for the fabrication of building elements with complex geometries that entail a significant degree of repeatability. Custom reusable formworks are designed and segmented to allow a directional demolding. A coating is necessary to prevent the wet concrete from bonding to the 3DPF surface. Reversible mechanical connections and release mechanisms between the formwork parts are also necessary. Reusable formworks need to be more robust and use more material to withstand the repeated handling and assembly. However, the high number of reuses can mitigate the higher production costs and result in a smaller cost per cast.

Relatively few projects have put forward and tested reusable 3DPF: Fast Complexity has reused two formwork modules twice for a slab prototype; the binder-jetted form liners of the Aufbaubank Stair can be reused up to 40 times; and the ABS formworks of the Domino Sugar Refinery building were reused 190 times. To ensure a good quality concrete surface and facilitate demolding, the three projects used a polyester gel coat. In addition, the formworks for the Domino Sugar Refinery building were machined to a precision of 0.12 mm to mitigate the material warp during fabrication and the aliased layered surface. These projects prove that reusability is not a technical constraint for 3DPF. However, in practice, reusability is limited by the nature of most 3DPF, which are intended for one of a kind, optimized concrete parts with no repetitive modules.

### Disposable formwork

Disposable formworks are usually removed through a destructive process once the concrete has cured, and are not intended to be reused to cast another identical piece. Conventionally, disposable formworks are used for complex, one of a kind concrete shapes, for which reusability is not required. The downside of disposable formworks is the wasteful nature of the process, and, implicitly, the high embodied energy. However, disposable formworks can become a viable construction method by using a minimum amount of material like in Submillimeter formwork. In general, polymer extrusion processes favor this type of minimal formwork shells because these also reduce the printing time significantly.

Another possibility is to use easily recyclable materials in closed-loop approaches. Ideally, in such cases, the material should be easily recyclable, like in the case of wax formworks.^68^ Dissolvable formworks, printed in PVA, which can be recrystallized and used to 3D print another set of formworks, have also been shown to work successfully.^53–56^ Clay, lacking water resistance, works similarly, requiring only water to be disposed of, and having the option of being recycled.

### Functional formwork

Functional formworks stay in place after the concrete has cured, and provide additional functionality to the concrete component, such as fire protection, acoustic properties, insulation, or surface finish. A conventional example is fire-retardant lost formwork used in tunnel construction.

Foam formworks are an example of functional 3DPF, being difficult to remove, but providing the thermal insulation function for the walls for the lifecycle of the building.^72^ Custom acoustic properties, targeting specific frequencies, can require precise and intricate surface textures, which can be provided by stay-in-place binder-jet 3DPF. This concept was proposed conceptually by The Smart Take from the Strong slab, but never tested.^76^ Optimized integrated building services can also be provided by 3DPF.^58^ Finally, stay-in-place 3DPF can serve to seal or confine the cast concrete, providing environmental protection against carbonation and freeze-thaw cycles. If made out of concrete, like in contour crafting, the formwork can provide a minimum concrete cover for the steel reinforcement, protecting it against environmental corrosion.

### Structural formwork

Structural formworks can be regarded as a subtype of functional stay-in-place formworks, but they contribute to the structural capacity of the concrete elements, usually by providing tensile reinforcement. Hence, structural formworks become an integral part of the reinforced concrete, acting together as a composite material. For this reason, structural formworks can be considered a standalone class of formworks.

Common types of structural formworks are based on ribbed sheet metal used for floor slabs, and textile reinforcement used for free-form shapes.^10,81^ The structural formwork stays in place after the concrete has cured and acts as axial or shear reinforcement.

Materials used in 3D printing generally lack the tensile strength of technical fabrics and steel, and are therefore unsuitable to provide principal reinforcement for concrete elements. Nevertheless, some thermoplastics such as nylon, as well as carbon-fiber reinforced polymers in general, have good tensile strength and can provide secondary tensile reinforcement and a durable finish. An example in this direction is Mesh Mold, where the spatially printed mesh functioned as secondary crack control reinforcement.^26^ A benefit of some polymers is their corrosion resistance, which means that they need no environmental protection when used for reinforcement, unlike steel.

### Permanent nonfunctional formwork

Permanent, lost, and stay-in-place formwork are terms often used interchangeably in the literature to refer to formworks that are not removed after the concrete has cured and may or may not perform functionally or structurally. To clarify the distinction from the other stay-in-place types, this class specifically refers to formworks that permanently stay in place after the concrete has cured, but do not provide any additional advantage for the building elements. In conventional construction, permanent formworks are always avoided, because they simply add dead load to the buildings, without bringing any benefit.

With 3DPF, permanent formworks may occur in instances where the formwork is trapped by or inside an inaccessible geometry. In these situations, the formwork needs to be minimized to reduce the resulting dead load added to the structure. The Smart Takes from the Strong slabs illustrate this approach, in which a binder-jetted 3DPF only 6- to 9-mm thick was used to cast the slabs and subsequently stayed in place, being trapped by the complex topology.

## Challenges

Conventionally, formwork designs need to take into account several challenges related to fabrication, assembly, removal, and end-of-life options. Out of all these, according to Peurifoy, the critical challenge remains safety.^82^ In conventional formwork systems, the safety challenges are generally associated with tolerances inherent in manual assembly and human errors. 3DPF systems are minimizing these risks, as they aim to be partly or even fully automated systems where fabrication tolerances and quality control are significantly better than in manual production. The design and precise digital fabrication of smart connection details can streamline assembly sequences and further reduce the risks associated with manual work.

Conversely, for digitally fabricated formworks, most challenges stem from the radical model that promises optimized, one-of-a-kind, and efficient concrete shapes with unique esthetics. These complex geometries are made possible by 3DPF, but present a significant challenge to fabricate and integrate the necessary reinforcement. 3DPF also struggle to compete with standard solutions in regard to their costs, time, and resources. In attempts to achieve cost-effectiveness by reducing material use, 3DPF can become fragile and inadequate to support the hydrostatic pressure forces during casting. This section will address these main challenges and discuss the solutions put forward in the literature.

### Robustness and hydrostatic pressure

A general challenge of 3DPF for tall objects like walls and columns is the hydrostatic pressure of the wet-state concrete. The hydrostatic pressure is the maximum stress exerted uniformly by the fluid concrete on the formwork. Hydrostatic pressure is only dependent on the density of concrete and the height of the column of fluid being cast, and it is independent of other dimensions of the object.

Especially, for disposable formworks, which are 3D printed minimally to reduce waste, the hydrostatic pressure can exceed the capabilities of the material, which lead to cracks and the sudden failure of the formworks. Brittle failures are especially problematic for the projects using fragile Submillimeter formworks. For these projects, there are several approaches to overcome the hydrostatic pressure that has been proposed in the literature.

The straightforward method is to increase the thickness of the formwork.^62,63^ Ribs and local stiffening geometries have also been proposed,^58,61,83^ as well as the use of strap systems for limiting deflections.^56,75^ Another simple approach that can be very effective is to rotate the object to minimize its height during casting, for example, to cast a column tilted horizontally. This approach was used in the MAS Stairs project.^59^

Using a counter-pressure material on the outer side of the formwork has also been successfully used to counteract the hydrostatic pressure of concrete. The approach involves placing the formwork in a container and simultaneously casting concrete inside the formwork, while adding the counter pressure material on the outside, in the container, making sure that the gradually increasing levels of the two materials are synchronized. Because the hydrostatic pressure is directly proportional to the density of the material, the counter-pressure material should ideally have a density close to that of concrete (∼2400 kg/m^3^ for regular concrete and usually ∼1400–1800 kg/m^3^ for lightweight concretes). However, even less dense materials can have a positive effect by partially offsetting the magnitude of the hydrostatic pressure. To this extent, the Concrete Canoe project has proposed the use of sand as counter pressure material, and Submillimeter formworks have suggested the use of water (Fig. 3).

Another method proposed in the literature is the sequential casting of concrete. Initially proposed by contour crafting,^33^ this approach was later employed in the Yhnova house, where concrete was cast in successive layers of 30 cm in height and allowed to build up load-bearing capacity before the next horizontal section was poured.^72^ Developing this idea a step further, Eggshell proposes a simultaneous 3D printing and casting process in which concrete is significantly accelerated for the hydration and structural buildup to occur within minutes after it is placed. The formwork only has the role of supporting the concrete for the first few centimeters, after which the fast structural buildup is eliminates the hydrostatic stress.^80^

### Reinforcement

A challenge of DDFC in general is the integration of adequate primary rigid steel reinforcement for structural applications.^6^ The additive, layer-based nature of the 3D printing process leads to collisions between the tool head and the prepositioned reinforcement. 3DPF overcome this issue because it relies on conventional concrete casting and decouples the digital fabrication process from the rebar positioning. In this sense, the fabrication sequence is not unlike a conventional casting process, in which the formwork is positioned first, followed by the insertion of the reinforcement, and finally the concrete casting. However, 3DPF still need to consider the geometric complexity, and more specifically, topological handles that can present accessibility issues and prevent rebar cages from being positioned in place. Such complex topologies demand complex reinforcement shapes as well, and to address this, several directions are being investigated.

Robotic bending, welding, and 3D printing can be used to fabricate reinforcement bars with complex geometries that can match the complex geometries of 3DPF.^84^ Steel and polymer fibers can be used, but only as secondary reinforcement for crack control.^68^ Recent studies have shown that microstructures designed in the surface of the formwork can influence the orientation of the fibers, to control the anisotropic properties favorably.^52^

Post-tensioning tendons can also benefit from the precision of 3D printing, and therefore several projects have already proposed the use of 3DPF for prestressed concrete.^59,79,83^ Usually, post-tensioning will not eliminate the need for rigid reinforcement entirely. Reinforcement strategies are therefore often combined because they complement each other: post-tensioning provides the primary bending reinforcement and rigid bar stirrups can provide shear reinforcement, while fibers or meshes provide secondary crack control reinforcement.^79,85^

Finally, due to the geometric flexibility of 3D printing, complex shells and slabs can be designed and fabricated to work in compression only.^86^ No tension forces occur within the concrete in such structures, and therefore no principal reinforcement is required.^87^ Unreinforced concrete is easier to fabricate and allows more complex shapes to be produced independent of reinforcement provisions. However, fiber reinforcement may still be necessary to handle shrinkage cracks.^58^

Other reinforcement strategies have been proposed for digital fabrication with concrete and complex shapes, but not necessarily for 3DPF. Nevertheless, some of these methods could be relevant and should be considered in future research. An external rigid or prestressing reinforcement system can bypass the intricate geometry and was demonstrated for the fabrication of a bridge with DDFC.^88^ Contour crafting suggests that flexible steel cables can be numerically deposited along with the extruded concrete filament, and this concept was tested in 2017.^89,90^ Such a system would be compatible with the sequential casting methods discussed previously.

Automatically placed meshes have also been proposed as an alternative to steel wires in a similar setup.^91^ Another method that could be compatible with sequential casting is the simultaneous reinforcement of concrete during 3D printing with overlapping staples.^92^ The opportunities and challenges of waterjet cut steel reinforcement have also been investigated in a series of small prototypes for topology-optimized concrete elements.^93^

Finally, metal 3D printing processes, such as wire arc additive manufacturing, can be used for the fabrication of steel structures.^94^ This is the process that is most closely related to 3DPF and promises the highest geometric freedom. The mechanical properties of 3D printed reinforcement and its bond behavior with concrete have been tested with satisfactory results.^95^ A preliminary test for this method with a mock-up material, steel powder-enriched PLA, has been tested in combination with dissolvable formworks 3D printed in parallel for a series of small-scale column prototypes.^54^

### Time and resources

3DPF are always benchmarked against conventional formwork solutions. Comparing modern prefabricated systems for mass production with 3DPF strictly regarding time and resources puts 3DPF at a significant disadvantage. Contemporary modular, standardized formwork systems can be installed very fast and efficiently and can be reused up to several hundred times with minimal maintenance, usually only needing to be washed, or the outermost sheathing layer replaced. 3D printing cannot rival with the advantages of mass production at this scale, at least not at the current state of the technology.

When comparing 3DPF to standard formworks, we need to account for the unique advantages of 3D printing, which are not compatible with conventional mass production. For example, for one-of-a-kind structures, for which reusability is not possible, disposable timber formworks are typically used. These require a specialized, wasteful, and laborious process. In such scenarios, 3D printing can compete with commercially available solutions, and it can even be more efficient. Efficiency, cost, time, and labor are essential questions for pioneers in the industry who aim to commercialize the process.

The FreeFab process, which was used for the fabrication of the custom concrete lining for the tunnels of three London Underground Stations, was considered up to 6.5 times faster than traditional formwork fabrication for the same elements.^68^ The ABS 3D printed and numerically milled formworks used by BAAM for the façade of a residential building in New York were 60% faster to make and only require 25% of the costs of conventional timber formwork.^63^ The 3DPF lining for the Aufbaubank Stair required 90% less assembly time for the formwork specialist.^78^ The Batiprint system can produce roughly 2 m^2^ of wall per hour, including the insulation layers.

These figures demonstrate that 3DPF can be very competitive in the context of other digitally fabricated formworks for bespoke concrete elements. Nevertheless, the challenge remains for the 3DPF to perform well also when compared to conventional fabrication of standard parts. A technical analysis published in 2020 concludes that polymer extrusion 3DPF are more than three times cheaper than conventional timber formworks and almost eight times cheaper than CNC milled formworks. The same study further assesses that polymer 3D printed formworks can be produced at a rate of 1.1 h/m^2^, which is about 14 times faster than conventional timber formworks and 9 times faster than CNC milled formworks.^96^ The fabrication times of 3D printing can be further offset by reusing the formworks and by selectively using the 3DPF for custom geometries only, while relying on more conventional formworks for the repetitive parts.^97^

The studies mentioned above base their figures on the price of materials, labor, and manufacturing, which results in promising figures. However, they fail to account for the costs and time necessary for designing, engineering, and preparing the parts for fabrication. While for standard prefabricated formworks, these costs are negligible, bespoke 3DPF need to be designed for every project.

Several other layers come into play when evaluating the price of 3DPF: reusable formworks are always ready to be delivered on site, whereas 3DPF have long production lead times; specialized training may be required for assembling and demolding them on site; a high initial investment cost might be necessary for specialized equipment, such as on-site robotic printers and concrete extruders; highly specific concrete mixes may be necessary; maintenance costs of reusable formworks may be higher; and so on. Conversely, 3DPF promise to make long-term savings by usually reducing the amount of concrete necessary and by ensuring more economical building lifecycles.

## New Possibilities

3DPF promise to make concrete architecture independent of fabrication constraints. However, the geometric freedom pursued by 3D printing is not a goal in itself. Instead, the goal is to enable new esthetic, functional, constructive, and structural possibilities with concrete.

**FIG. 9.**
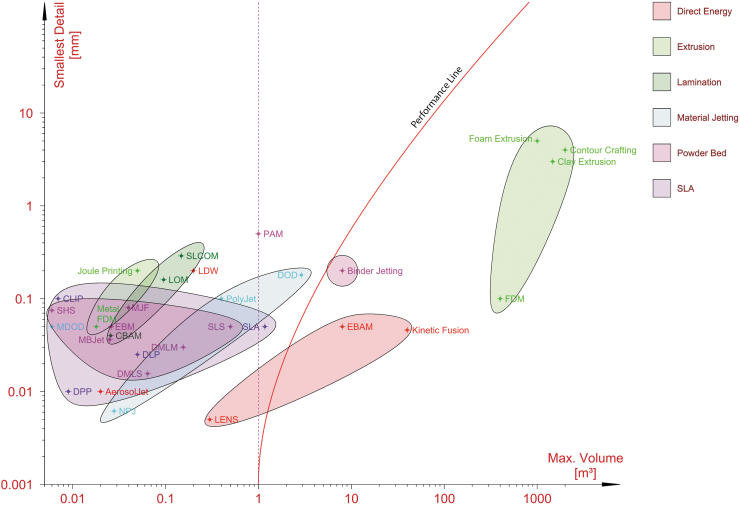
An Ashby plot of the estimated smallest feature size on the y axis and build volume on the x axis of the most common 3D printing technologies. The performance line separates the technologies with adequate properties for use as formworks to the right. The data are compiled from available commercial specification sheets from one to five manufacturers for each of the following technologies: clay extrusion, composite-based additive manufacturing (CBAM), continuous liquid interface production (CLIP), contour crafting, daylight polymer printing (DPP), digital light processing (DLP), direct metal laser melting (DMLM), drop on demand (DOD), electron beam additive manufacturing (EBAM), electron beam melting (EBM), foam extrusion, fused deposition modeling (FDM), Joule printing, kinetic fusion, laminated object manufacturing (LOM), laser direct writing (LDW), laser-engineered net shaping (LENS), multijet fusion (MJF), nanoparticle jetting (NPJ), poly-jet, precision additive metal manufacturing (PAM), selective heat sintering (SHS), selective lamination composite object manufacturing (SLCOM), selective laser sintering (SLS), stereolithography (SLA). (© DBT, ETH Zürich, 2019).

3D printing allows more geometric freedom than subtractive and formative processes. However, the various 3D printing processes have different specific geometric limitations and constraints. Fig. 9 compares the commercially available 3D printing processes in an Ashby scatter plot typically used in material selection, mapping two competing properties.^98^ The x axis maps the size of the smallest stable geometric feature that can theoretically be fabricated (R), the inverse of resolution. The y axis maps the maximum build volume (V). The data are compiled from available commercial specification sheets obtained from manufacturers. One to five different manufacturers are cataloged for each of the 30 different technologies. Assuming a minimum volume (V_m_) of at least 1 m^3^ is required, the performance index (P_vr_) can be expressed as follows:

(1) $$P_{vr}=f\left(\frac{V}{R-V_{m}}\right).$$

Taking the log of both sides and rearranging the terms, this gives

(2) $$log\left(V\right):=log\left(P_{vr}\right)+log\left(R-V_{m}\right).$$

For *V*_m_ = 1, (2) is an equation of the form y = p × (x − 1), which is a parabola with the apex in *x* = 1 in a log–log plot. The desirable processes are found to the right of this performance curve.

The graph highlights to the left of the performance curve the 3D printing processes that are not suitable due to their limited print bed sizes: material jetting, lamination, stereolithography, and metal filament printing. These would require extensive discretization for large formwork parts and implicitly, puzzles with many pieces, difficult to assemble.

Extrusion-based processes and binder jetting are situated to the right of the performance curve, and therefore they enable a significant improvement in geometric complexity for concrete at large scale. With these processes, the formwork is no longer a bottleneck for the geometric complexity of concrete. The geometric limitations are now imposed by other constituents of concrete construction, such as provisions for reinforcement fabrication and placing, accessibility for casting, rheology of concrete, size of the aggregates, concrete processing, and directional demolding.

Due to their excellent geometric freedom, 3DPF can have significant implications, beyond simple surface effects. 3D printing can open new design possibilities for more elaborate free-form geometries, organic shapes, porosity gradients, complex topologies, microtextures, and high-resolution ornamental surface articulation in concrete. 3D printing bridges the gap between the unlimited design freedom *in silico* and the reality of physical fabrication. The Smart Slab demonstrates the capabilities of 3DPF well, exhibiting a convoluted, ornamented surface with digitally designed organic features with varying amplitudes and widths in the range of only a few millimeters. The Smart Slab further demonstrates how a complex macro topology, with openings above the supporting wall, allows natural light to penetrate deep inside the space, thus contributing to the overall atmosphere of the unit (Fig. 6).^77^

### Surface textures

Conventional formwork has only limited esthetic implications for architectural concrete. Exposed form tie marks and segmentation seams can be visible traces of the fabrication process. Furthermore, sometimes rough, smooth, satin, or glossy textures can be controlled through the formwork, form lining, coating, and release agents. In contrast, 3D printed parts typically exhibit a distinctive layered surface texture inherited from the layer-based manufacturing process (Fig. 2). This surface effect is faithfully transferred to the concrete part, regardless of the size of the layers. Submillimeter layers, like in polymer extrusion (0.2 to 0.4 mm) and binder jetting (ca. 0.3 mm), result in smooth surfaces and clean topological lines.

On the other hand, concrete, foam, and clay 3D printing result in clearly distinct layers with heights varying from 5 to 20 mm, which are often irregular and populated with fabrication artifacts, which are especially irregular in foam 3D printing.

To address these issues, researchers have proposed several postprocessing methods for the 3DPF. Gel coatings reduce the layered effect and have been used in polymer extrusion and binder jetted formworks, but result in rounded inner edges.^83^ Polymer extrusion 3D printing has also been shown to respond well to chemical treatments with solvents, heat treatments, and mechanical ball milling.^62^ Foam and polymer formworks have also been used in combination with CNC milling to adjust edges and surface quality.^63^ Finally, it is also possible to mechanically process the resulting concrete part to achieve a smooth surface quality, but this process is energy-intensive and is not compatible with fiber-reinforced concrete.^38^

Alternatively, the layered esthetic has also been embraced as a design intent and esthetic expression of the fabrication process. This layering effect is embraced by projects attempting to produce clean, regular layer contours, as well as projects that actively stimulate the occurrence of fabrication artifacts and embrace their expressive esthetic.^99^

### Functional integration

3DPF are often used in stay-in-place scenarios, due to the complex geometries with interlocking features, which make removal difficult. Stay-in-place formworks often integrate an additional function that justifies the added dead load to the building. Functional integration is particularly interesting in combination with stay-in-place formworks that can act as fire or thermal insulation or can improve acoustic properties (Fig. 8).^76^ Another example of specialized functional integration enabled by 3DPF is exhibited by the MAS Stair prototypes that feature a corrugated antislip stepping surface.^59^ Anti-slip surfaces are typically provided by an auxiliary metal or plastic nosing that is difficult to tailor for bespoke stairs.

Thermal insulation is the only type of integration that has already been investigated in depth and prototyped in large scale. The Yhnova system is a sandwich wall with two 80-mm-thick external layers of foam and a 150-mm-thick concrete core that achieves a thermal resistance of 6.75 m^2^K/W, which can reduce the energy consumption in a prototypical house by 30 to 40%.^72^ These are comparable results to the more standard Insulated Concrete Formwork (ICF) construction, which consists of lightweight hollow insulating interlocking blocks that act as formwork for reinforced concrete, and can achieve between 5 m^2^K/W (Logix ICF panels) and 6.67 m^2^K/W (BASF Neopor with graphite), depending on the material used for the insulation.

### Integration of services

The integration of services in building elements originates from lean manufacturing, a concept borrowed from the automotive industry, where components are preassembled with all the necessary subcomponents and then put together in assembly lines. This approach increases productivity, reduces waste, and facilitates serviceability and incremental improvements.

In buildings, this approach implies the integration of building services, such as electrical conduits, HVAC ducts, light fittings, and fire sprinklers, in the prefabrication stage, rather than fitting them on site. This approach ensures better quality control and reduces construction time on-site. Nevertheless, there are concerns regarding the serviceability of integrated building systems, and the shorter life cycles of building services (15–20 years), as opposed to structures (50 years).^100^

With the complex geometries facilitated by 3DPF, standard off-the-shelf parts are not adequate for the necessary building services. In these instances, building services can be integrated in the structural concrete and custom designed to fit the complex geometry. The custom design opens an opportunity toward efficient and optimized building services, tailor made for the particular space to reduce the amount of energy.

3DPF can facilitate a partial integration, in which only the provisions, voids, and openings for building services are defined in the concrete volume, while the ducts and pipes are installed after casting. Lighting features, electrical conduits, and water pipes were integrated in this way by projects such as the NowLab Wall and the Smart Slab.

A further step in integration relies on the stay-in-place formwork itself to act as air duct or cable tray. This full integration concept was demonstrated in the Funicular Slab, in which the integration of an innovative chilled beam HVAC system was enabled by polymer extrusion 3DPF (Fig. 5).^58^ The stay-in-place thin plastic formwork defined two inlets: a duct bringing in fresh air and an internal inlet directed through a heat exchanger. Six air outlets were also defined to distribute the flow of air uniformly toward the internal space. The shape of the network of ducts was optimized through CFD software to minimize air pressure loss. The resulting geometry could not have been fabricated with standard off-the-shelf parts.

### Automation for mass customization

Typically, formwork manufacturing for bespoke parts requires a relatively large component of labor. Some 3DPF also require a series of postprocessing steps to prepare them for concrete casting. Binder jetting is a typical example, in which process-specific desanding, as well as infiltration and coating steps are necessary for stabilizing the parts and for ensuring a smooth surface. Nevertheless, processes like contour crafting, BatiPrint, clay formworks, and to a certain extent polymer 3DPF produce ready-to-use formworks that can be cast straight away. Such an example is the Eggshell formwork, for which the fabrication of formwork and casting are done simultaneously.^57^

Digital design and fabrication have the potential to automate construction fully. Computational design tools can integrate all the project-specific processes, such as segmentation for prefabrication, formwork design to fabrication, toolpath generation, collision detections, demolding, and construction sequencing. Recently, computational methods for developing functional molds for complex geometry have been developed, focusing on discretization methods, geometric approximations of free-form geometries for casting, and calculating optimal demolding directions.^101–105^

The implications of this automation opportunity are beyond the typical scenario of an unsupervised production line. Digital fabrication allows cost-effective mass customization and production, in which each piece of formwork can be unique, allowing the concrete components to respond optimally to their spatial, structural, and functional conditions. Mass customization is demonstrated by BigRep, who propose in their patent the gradual variation of formwork thickness, in response to the height-dependant hydrostatic pressure.^61^ The Smart Slab further demonstrates mass customization, illustrating it through 11 similar, but unique segments that each respond efficiently to the asymmetrical loading conditions (Fig. 6).

## Discussion

Historically, the main driver in formwork innovation has been to improve cost efficiency through modularity, reusability, serialization, rationalization, and preassembly. The most significant innovations in formwork construction focus directly on improvements of the construction process that have quick commercial benefits. Any improvement of the concrete structure itself is generally incidental, indirect results of the formwork systems innovations. Some of the major innovations in the field of formwork include Sequential Molds,^106^ Self-Aligning Molds,^107^ Mold Carriers,^108^ Tunnel Formwork,^109^ Slip Forming,^110^ Flying Formwork,^111^ Sliding Formwork,^112^ Self-Climbing Formworks,^113^ Insulated Concrete Forms,^114–116^ Self-Supporting Forms, Hinged Formwork,^117,118^ Battery Formwork,^119^ and Butterfly Formwork.^120^

These efficient and simple-to-use formwork systems are up to three times more expensive than conventional formwork. However, they result in savings in labor and a remarkable increase in productivity, as well as important financial savings from reusability, with the most performant systems reaching more than 700 concreting cycles with minimal downtime for maintenance.^121^

### Selecting the right 3D printing method

The new 3DPF systems reviewed in this article generally contradict the previous trend of innovative commercial formwork systems. 3DPF are generally disposable or limited-reuse systems and aim to enable bespoke optimized structures and one-of-a-kind designs, tailored for a specific context, rather than mass-produced building element serialization. Although the early attempts are expensive, less robust, and time-consuming, they can also potentially become commercially competitive through automation.

Some types of building elements have specific suitable 3D printing technologies. The hydrostatic pressure of concrete during the casting of tall parts is one of the critical considerations. Slabs and horizontal concrete elements, such as beams and arches, generate the smallest stresses on the formwork, and therefore, binder jetting and polymer extrusion have been favored due to their excellent surface quality. For tall elements, such as columns and walls, concrete and foam extrusion have been the most successful methods, due to their higher strength. Polymer 3DPF through the Eggshell and Dissolvable PVA approaches have also successfully produced objects up to 3 m in height. Finally, intrinsically complex elements, such as stairs and space frames, are generally more suitable for polymer extrusion and binder jetting, technologies that enable more geometric freedom than the other extrusion processes.^59^

### Digital concrete or digital formwork

Are 3DPF just a stepping stone along the way to fully integrated form-free DDFC? Research has shown that 3DPF are ready for implementation in load-bearing building elements. In contrast, the limitations of reinforcement are still far from being resolved for DDFC. Even projects that claim to have fully 3D printed entire buildings are in effect 3DPF made of concrete in most cases.^35^ There are, however, niche applications for concrete extrusion that can be implemented sooner, such as nonstructural elements and compression-only structural systems.

### Sustainability

Two aspects concerning the sustainability of 3DPF need to be considered: the sustainability of the formwork itself, and the indirect impact on the sustainability of the resulting concrete parts.

#### Sustainability of 3DPF

Among the 3DPF materials, concrete and clay perform very well due to their minimal specific embodied energy, while at the other end of the spectrum, foams, plastics, and organic binder sandstone have embodied energies higher than steel. The main handicap of most 3DPF systems is their intended one-time use. For unique parts, each cast inherits the entire energy footprint of the formwork.

With careful designs and adequate postprocessing and coating, reusability is a viable solution. However, so far, there is limited research in this direction. The material generally used for coating polymer and binder jet formwork is polyester, which provides a smooth and mechanically and thermally durable surface, also used as a matrix for the fabrication of GFRP. GFRP formworks can achieve upwards of 200 reuses with little to no maintenance work in between casts. This figure has been confirmed by BAAM, who coated ABS 3DPF and reused them 190 times without reaching their end of life.^122^

One way to mitigate the high embodied energy per cast of one-time-use 3DPF is to minimize the formwork thickness (Fig. 3).^51^ Furthermore, minimal 3DPF can be combined with standard reusable scaffolding systems for stiffness.^97^ Another strategy to alleviate the adverse impact of one-time use is the integration of the formwork in the permanent architecture, as a functional, structural, or aesthetic constituent part of the building. For example, foam formworks achieve this by essentially doubling as permanent insulation for concrete walls.

#### Material efficiency and sustainability of concrete parts

The embodied energy of disposable 3DPF is measurably higher compared with reusable formwork systems. However, 3DPF can have a further indirect impact on the embodied energy of the concrete parts, as well as on the long-term operational energy of buildings overall. The indirect benefits stem from the material-efficient designs based on topology optimization enabled by 3DPF. Several projects have demonstrated how the weight of slabs can be reduced by up to 70% (Fig. 8).^58,76,77^ This reduction in concrete use outweighs the increase in embodied energy coming from the 3D printing processes.

The second important aspect is the saving in operational energy of a building that can be achieved through smart geometries. Operational energy can constitute up to 80% of the total lifecycle energy of a building, and therefore even small improvements can make a big impact. Optimized building systems integrations, efficient HVAC systems, as well as spatial and material savings from integrating services within the structural bounding box of building elements, can all have a positive impact on the operational energy of a building. The integration of such systems has already been demonstrated in the Funicular Slab.^58^ The functional hybridization has been shown to shorten the lifecycle of a building, but even taking this into account, the integrated concrete elements still outperform conventional components regarding their operational energy.^100^

## Conclusion

3DPF challenge the status quo in which rationalization for mass production and modular reusable assemblies are dominating the market to cut costs. 3DPF can be used in stay-in-place, *in situ*, or disposable strategies, approaches that are generally avoided in commercial formwork systems due to their incompatibility with the economy of scale. However, 3DPF projects manage to reinvent the formwork typologies and put forward clever solutions to construction challenges. These new formwork typologies trigger more freedom in design thinking and allow novel architectural solutions to be implemented.

**FIG. 10.**
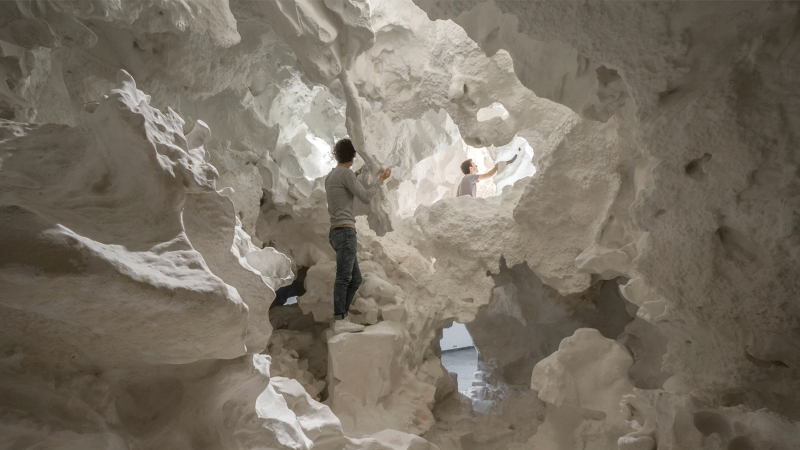
The Incidental Space pavilion of Switzerland at the Venice Biennale in 2016 illustrates a radical interpretation of architectural space that is not based on archetypical building elements such as beams and columns. The pavilion was fabricated with digitally fabricated formworks, partly milled and partly binder-jet 3D printed (Christian Kerez/DBT, ETH Zürich, 2017).

Radical creative solutions based on 3DPF can challenge fundamental principles of architectural design, such as the archetypical building elements and the right angle. The ubiquity of the right angle in architecture is partly a result of fabrication constraints, which no longer apply to 3D printing. The Incidental Space is a relevant example in this direction, where conventional building elements blend into a continuous shell that illustrates not only a radically new esthetic but also a viable structural solution (Fig. 10).^75^

### Outlook

The analysis of the state-of-the-art presented in this article has shown that the available hardware is one of the major limiting factors for 3DPF. Technological advancements should focus on providing faster fabrication times, more robust and reliable processes, and more sustainable materials suitable for architecture. New research streams are expected to cover other 3D printing methods that are relevant at large scale, such as stereolithography and metal welding 3D printing, as highlighted by the Ashby plot in Figure 9. In particular, kinetic fusion is an emerging technology that can fabricate volumes of 40 m^3^ at up to 75 kg of metal per hour. While the costs of this technology are still relatively high to justify single-use formwork fabrication, it is promising for custom rigid reinforcement cages that could synergize with 3DPF. Other hybrid methods are also expected to follow up, combining 3D printing with complementary digital or nondigital fabrication methods.

Furthermore, a critical research topic in this field should continue to be sustainability, both for formworks and the resulting concrete parts. Reusable 3DPF are underrepresented in current research. Along with the development of new sustainable and recyclable materials suitable for disposable 3DPF, reusability can have a positive impact on the sustainability of concrete constructions.

Finally, the potential of stay-in-place formworks needs to be explored further as well. Initial research projects have mapped out possible performative aspects that can be covered by 3DPF, such as fire protection, acoustic diffusion, and radiant heating and cooling. However, these aspects should be further explored quantitatively in full scale prototypes.

### Contribution

Beyond their direct implications for the construction sequence, formworks can leave their mark on architecture on a deeper semantic, aesthetic, and functional level. This article has shown how different types of formwork have meaningful architectural implications on the possible geometries, structural schemes, building services, functionality, esthetics, surface quality, and finishes.

This article further mapped the domain of 3DPF in both academic research and pioneering industry endeavors. 3DPF have already been investigated for more than 25 years, and research has matured from early patents and small-scale experiments to prototypical pavilions and finally to functional large-scale architectural applications. While recent projects demonstrate a clear potential in commercial architecture and infrastructure projects, it has so far only been used in test applications.

3DPF have to compete with commercial solutions that are currently faster, more robust, reusable, adjustable, modular, and relatively flexible within a certain standardized typology. However, these often result in oversized components due to the lack of versatility, and sacrifice variety and design freedom for resource economy. On the contrary, 3DPF enable bespoke, optimized building elements that are already being used occasionally for one-of-a-kind showcase buildings. Weight reduction, sustainability, functional integration, a new esthetic, and automation are the key opportunities of 3DPF for concrete that are difficult to achieve otherwise.
